# Preparation, characterization, and anticancer effect of Capsaicin-functionalized selenium nanoparticles

**DOI:** 10.3389/fnut.2024.1515657

**Published:** 2024-12-20

**Authors:** Enhui Tang, Ziqing Ma, Peiting Zhang, Yuyang Chen, Yiman Zhou, Jieying Wu, Tingting Yang, Duanya Lian, Xinlan Wu

**Affiliations:** ^1^School of Public Health, Guangzhou Medical University, Guangzhou, China; ^2^School of Anesthesiology, Southern Medical University, Guangzhou, China

**Keywords:** Capsaicin, selenium nanoparticles, anticancer, apoptosis, HepG2

## Abstract

**Introduction:**

Selenium nanoparticles (SeNPs) are recently emerging as promising anticancer agents because of their high bioavailability, low toxicity and remarkable anticancer activities. However, the application of SeNPs in anticancer has been limited due to instability. Herein, Capsaicin (Cap), a natural active compound found in chili peppers with favorable anticancer activity, was modified with SeNPs to prepare Cap-decorated SeNPs (Cap@SeNPs), and the antiproliferative effect and mechanism of Cap@SeNPs in HepG2 were investigated.

**Methods:**

Cap@SeNPs were prepared through a redox method and characterized using ultraviolet-visible spectroscopy and Fourier transform infrared spectroscopy. Subsequently, the inhibitory rate of Cap@SeNPs on HepG2 cells was determined by the MTT assay. Finally, the antiproliferative mechanism of Cap@SeNPs was explored through analysis of cell cycle, cell viability, reactive oxygen species levels, mitochondrial membrane potential, nuclear morphology, and caspase activity.

**Results:**

Our results revealed that stable and well-dispersed Cap@SeNPs were successfully fabricated, and the optimum mass ratio of sodium selenite to Cap was 1:2. In addition, Cap@SeNPs showed significant antiproliferative effects on HepG2 cells compared with naked SeNPs. Furthermore, Cap@SeNPs inhibited the proliferation of HepG2 cells by elevating total ROS levels, causing nuclear condensation, affecting mitochondrial membrane potential, which in turn influences caspase protease activity and induces apoptosis.

**Conclusion:**

This study developed an innovative approach to enhance the value of Cap, demonstrating that Cap@SeNPs hold promise as potential therapeutic agents for cancer treatment.

## 1 Introduction

Currently, cancer is one of the major causes of human mortality, posing a serious threat to human life and health. Liver cancer is ranked as the sixth most common cancer and the third leading cause of cancer-related deaths worldwide (1). According to the International Agency for Research on Cancer (IARC) GLOBOCAN cancer statistics, 83,000 people died of liver cancer in 2020, second only to lung cancer (1.79 million deaths) (2). Some of the traditional and newer methods of treating cancer, such as surgical resection, radiation therapy, chemotherapy, targeted therapy, and immunotherapy have relevant side effects that negatively impact the quality of life (3). For instance, chemotherapy drugs are highly cytotoxic and also kill normal cells during treatment (4). Thus, the development of novel drugs with high selectivity and low cytotoxicity is important for cancer treatment. In recent years, metallic nanoparticles have been a step toward more effective cancer treatment strategies, which are capable of simultaneously encapsulating and administering multiple anticancer drugs with different therapeutic mechanisms, making them ideal candidates for cancer therapy (5).

Selenium (Se), an essential micronutrient for humans, is vital to the health of the organism (6). Se is a component of the amino acid selenocysteine, which produces selenoproteins, including the antioxidant enzyme glutathione peroxidase (GPx) (7). Furthermore, Se is involved in metabolism, regulation of the immune and reproductive systems, DNA synthesis and apoptosis, resistance to oxidative damage, and development and regeneration of muscle tissue (8, 9). Both deficiencies and excesses of Se can affect the physiological functions in humans (10). Due to the narrow range of Se physiological requirements and toxic doses, the rational and active application of Se in the control of human diseases and health remains a great challenge (11). In recent years, with the development of nanotechnology, Se nanoparticles (SeNPs) possess lower toxicity and higher bioavailability compared to organic or inorganic forms of Se compounds (12). SeNPs present attractive anti-cancer activity and have been used to treat a variety of diseases such as diabetes, inflammatory diseases, liver fibrosis, and drug-induced toxicity (13, 14). However, nano-sized Se is very unstable and prone to spontaneous aggregation into clusters and precipitation, resulting in reduced bioactivity and bioavailability (15).

In recent years, researchers have explored various stabilizers to overcome the instability of SeNPs and enhance their bioavailability and bioactivity. These stabilizers are broadly classified into inorganic materials, organic polymers, and natural biomolecules. Inorganic materials like silicon dioxide and alumina can protect SeNPs through physical coating (16). Organic polymers, such as polyethylene glycol (PEG), are commonly used due to their water solubility and biocompatibility (17). Natural biomolecules, including polysaccharides, proteins, and plant chemicals, have gained attention due to their renewability, low toxicity, and biocompatibility. Polysaccharides like chitosan and starch, and proteins like bovine serum albumin (BSA), form stable complexes with SeNPs through hydrogen bonding and electrostatic interactions (18). Plant chemicals, like betacyanins, enhance the antioxidant and anticancer properties of SeNPs (19). These natural molecules improve SeNPs' stability and retain or enhance their bioactivity. However, these stabilizers have limitations: inorganic materials may be toxic, organic polymers may obscure bioactive sites, and polysaccharides and proteins may show inconsistent stability due to differences in their sources and batches (18, 19). Therefore, plant chemicals have received significant attention. Currently, it is particularly important to identify more novel stabilizers from plant chemicals that can effectively stabilize SeNPs while retaining or enhancing their bioactivity.

Capsaicin (Cap)(trans-8-methyl-N-vanillyl-6-noneamide), also known as trans-8-methyl-N-vanillyl-6-noneamide, is a lipophilic bioactive alkaloid naturally extracted from *Capsicum spp*. Cap has a wide range of biological activities such as anti-inflammatory, antioxidant, anti-obesity, and improvement of circadian rhythm disorders (20, 21). Moreover, Cap showed potent anti-cancer activity as demonstrated by playing an anti-cancer role *in ex vivo* experiments related to nasopharyngeal, lung, breast, gastric, liver, colorectal, and prostate cancers (22, 23). Furthermore, Cap can selectively inhibit the growth of tumor cells and has little effect on the proliferation and differentiation of normal cells (such as adenocytes and hepatocytes) (24). Nevertheless, the clinical application of Cap as a viable anticancer drug remains problematic due to poor water solubility and low bioavailability (25). To improve the bioavailability of Cap, researchers have prepared Cap-loaded nanoemulsions (26), self-assembled Cap prodrug nanoparticles (27), and Cap-loaded nanoliposomes (28), etc. Based on the molecular structure of Cap and the chemical properties of Se, it is of great significance to prepare Cap-selenium nanocomposites by combining Cap as a surface modifier with SeNPs, which not only reduces the free energy on the surface of SeNPs and enhances their stability, but also improves the bioavailability of Cap. However, the study of combining Cap as a surface modifier with SeNPs to utilize their synergistic anticancer effects has not yet been reported.

In the present study, the Cap-decorated SeNPs (Cap@SeNPs) were prepared by capping Cap onto the surface of SeNPs and their *in vitro* anticancer effects in HepG2 cells were investigated subsequently. Furthermore, the mechanistic pathways of apoptosis in HepG2 cells caused by Cap@SeNPs were also studied by evaluating the level of oxidative stress, mitochondrial membrane potential alteration, and activation of caspase proteases.

## 2 Materials and methods

### 2.1 Materials

Cap and Methyl thiazolyl tetrazolium (MTT) was purchased from Sigma-Aldrich Co. (St. Louis, MO, USA). L-ascorbic acid (analytical grade) and sodium selenite (Na_2_SeO_3_; purity ≥ 98%) were purchased from Rhawn Chemical Technology Co. (Shanghai, China). Fetal bovine serum (FBS), penicillin-streptomycin solution, Dulbecco's modified Eagle's medium (DMEM), pancreatin-EDTA solution (0.25%), and phosphate-buffered saline (PBS) buffer solution were purchased from GIBCO (Waltham, MA, USA). Reactive oxygen specific (ROS) assay kit and mitochondrial membrane potential assay kit (JC-1) were purchased from Beyotime Biotechnology Co. (Shanghai, China). Hoechst 33258 dye was purchased from Beijing Lanjieke Technology Co., Ltd. (Beijing, China). BCA protein assay kit, RIPA lysis buffer, and protease inhibitor cocktail (EDTA-free, 100 × DMSO) were purchased from Biosharp. Ac-DEVD-AMC for caspase-3, Ac-IETD-AFC for caspase-8, and Ac-LEHD-AFC for caspase-9 were purchased from Macklin Biochemical Co., Ltd (Shanghai, China), Med Chem Express LLC (Rahway, NJ, USA), and *Kekule* Biomedical Co., Ltd. (Guangzhou, China), respectively. FITC Annexin V apoptosis detection kit with PI was purchased from Qihai Biomedical Technology Co., Ltd. (Shanghai, China). Z-VAD-FMK was purchased from Biyuntian Biomedical Technology Co., Ltd. (Shanghai, China).

### 2.2 Preparation of nanoparticles

The preparation method of nanoparticles referred to a previous study with some modifications (29). Stock solutions of 0.87 mg/mL Na_2_SeO_3_ [dissolved in double-distilled water (ddH_2_O)], 3.52 mg/mL ascorbic acid (dissolved in ddH_2_O), and different concentrations of Cap (dissolved in anhydrous ethanol) were prepared, respectively. Subsequently, 5 mL Na_2_SeO_3_ solution was mixed with 5 mL Cap solution, and 5 mL ascorbic acid solution was added into the mixture slowly and dropwise under sonication. Then, the mixture was diluted to 25 mL with ddH_2_O. Until the red color of the suspension no longer deepened, the mixture was moved to the refrigerator at 4°C and continued to react for 24 h. For the preparation of pure SeNPs, the Cap solution was replaced with the same volume of ddH_2_O following the procedure above. After that, the suspension was transferred to a 3,000 Da dialysis bag and dialyzed for 24 h under magnetic stirring to remove the excess Cap, ascorbic acid, and selenite. Subsequently, the particle size and zeta potential of the samples were determined by a Zetasizer Nano ZS instrument (Malvern Instruments Ltd, UK).

### 2.3 Morphology analysis of nanoparticles

The suspension of SeNPs and Cap@SeNPs were lyophilized to powder using a freeze-dryer (Ningbo Xinzhi Biotechnology Co., Ltd, Zhejiang, China), and stored in a desiccator at room temperature before use. Prior to scanning electron microscope (SEM) observation, the conductive adhesive was first evenly applied to the sample holder. Subsequently, appropriate amounts of powder samples were deposited onto the adhesive-coated sample holder, ensuring an even distribution to mitigate any agglomeration. This was followed by gold sputtering of the samples to bolster the contrast in SEM imagery. Ultimately, the appearance of nanoparticles was observed through a Phenom ProX scanning electron microscope (Funa Scientific Instruments Co., Ltd., Shanghai, China).

### 2.4 Ultraviolet-visible absorption spectrum analysis

0.5 mg/mL of Cap, SeNPs, and Cap@SeNPs solutions were configured, respectively. The UV-vis absorption spectra were recorded by a UV-2600 spectrophotometer (Shimadzu, Kyoto, Japan) with a wavelength range of 200–800 nm.

### 2.5 Fourier transform infrared spectrum analysis

10 mg of Cap, SeNPs, and Cap@SeNPs powders were mixed and ground with KBr at a ratio of 1:100 (w/w), respectively. The FT-IR spectra of Cap, SeNPs, and Cap@SeNPs were recorded by a Thermo Nicolet NEXUS 670 FTIR spectrometer (Thermo Scientific, WestPalm Beach, USA) with a frequency range and resolution of 400 to 4,000 cm^−1^, respectively.

### 2.6 Cell culture

The HepG2 and LO2 cell line were cultured in a mixture of FBS (10%), penicillin and streptomycin solution (1%), and DMEM. HepG2 or LO2 cells were grown in T25 cell culture flasks at 37°C and 5% CO_2_ in an incubator.

### 2.7 Cell viability assay

Cell viability of HepG2 was assessed using the MTT assay. Briefly, cells were seeded in 96-well plates at a density of 2 × 10^4^ cells/well and incubated for 24 h. Subsequently, cells were treated with different concentrations of Cap, SeNPs, and Cap@SeNPs, and allowed to grow for 12, 24 and 48 h. After that, 100 μL of 0.5 mg/mL MTT solution was added to each well, with care taken to operate in the dark. After 4-h incubation at 37°C, the supernatant is discarded, and 100 μL of DMSO was slowly added to each well using a pipette. The solution is then gently shaken for 10 min at a low speed. Absorbance was measured at 570 nm by a Multiskan 60 microplate reader (Thermo Fisher Scientific, Waltham, MA, USA), from which the cell inhibition rate and the half maximal inhibitory concentration (IC50) of the sample are calculated. The morphology of HepG2 cells was observed by the Nikon Eclipse E100 upright optical microscope with the NIKON DS-U3 imaging system (Nikon, Japan).

### 2.8 *In vitro* cytotoxicity of Cap@SeNPs

LO2 cells were plated in 96-well plates at a density of 2 × 10^4^ cells per well and allowed to incubate for 24 h. Following this, the cells were exposed to varying concentrations of Cap@SeNPs and Selenite, and were further incubated for periods of 24 and 48 h, respectively. Ultimately, cell viability was evaluated through the MTT assay, as detailed in Section 2.7.

### 2.9 Cell cycle analysis

HepG2 cells were seeded in six-well plates at a density of 4 × 10^5^ cells/well and incubated for 24 h. After 24 h of treatment with different concentrations of Cap@SeNPs (at 30, 60, and 120 μg/mL), cells were collected and fixed in 75% ethanol overnight at 4°C. Subsequently, cells were stained with PI dye (with RNase A) and incubated for 30 min at room temperature. The cell cycle distribution of HepG2 was analyzed by the AccuriC6 flow cytometer (BD Biosciences, NJ, USA).

### 2.10 Cell apoptosis analysis

HepG2 cells were seeded in six-well plates at a density of 4 × 10^5^ cells/well and allowed to grow for 24 h. After 24 h of treatment with Cap@SeNPs at 30, 60, and 120 μg/mL, cells were collected and stained with Annexin V-FITC and PI dye according to the manufacturer′s instructions. The apoptosis distribution of HepG2 cells was determined by the BD AccuriC6 flow cytometer. During experiments pertaining to the caspase inhibitor Z-VAD-FMK, cells were first pretreated with Z-VAD-FMK at a concentration of 20 μM for a period of 1 h. Following this pretreatment, the Cap@SeNPs was introduced to the cells for concurrent interference. The overall percentages of apoptotic cells were determined by including the cells located in both the lower and upper right quadrants (30).

### 2.11 Intracellular ROS level assay

In this work, HepG2 cells were stained with the DCFH-DA (10 μM) dye as the fluorescence probe and incubated at 37°C for 30 min. Following treatment with Cap@SeNPs at 30, 60, and 120 μg/mL for 30 min, the intracellular ROS levels were determined by a microplate reader with excitation and emission wavelengths at 488 and 525 nm, and the stained cells were observed using fluorescence microscopy (Nikon Ts2-FL, Tokyo, Japan) with excitation and emission wavelengths at 495 and 525 nm.

### 2.12 Mitochondrial membrane potential (ΔΨm) assay

For flow cytometric analysis, HepG2 cells were treated with different concentrations of Cap@SeNPs at 30, 60, and 120 μg/mL for 24 h, washed with PBS, harvested by trypsinization, and stained with JC-1 working solution according to the manufacturer′s instructions for 10 min. The ΔΨm of HepG2 cells was measured by the flow cytometer. Similarly, after 24 h of treatment with Cap@SeNPs, the cells were washed with PBS and stained with JC-1 dye for 10 min at 37°C. Subsequently, the stained HepG2 cells were observed by a fluorescence microscope, and the fluorescence intensity was determined by a microplate reader.

### 2.13 Nuclear morphology observation

After 24 h of treatment with Cap@SeNPs (at 30, 60, and 120 μg/mL), HepG2 cells were washed with PBS and fixed in 4% methanol for 10 min at 4°C. The cells were then stained with Hoechst 33258 dye for 10 min at 37°C, followed by washing with PBS. The fluorescence intensity of stained cells was recorded using a fluorescence microscope.

### 2.14 Caspase activity assay

HepG2 cells treated with Cap@SeNPs at concentrations of 30, 60, and 120 μg/mL for 24 h were washed with PBS and collected by trypsinization. Subsequently, the cells were resuspended with RIPA lysis buffer and incubated on ice for 30 min at 4°C. After centrifugation at 1,200 × *g* for 15 min, the supernatant was collected and the protein concentration was immediately determined using a BCA kit. After that, the cell lysates were placed in 96-well plates and specific caspase substrates (Ac-DEVD-AMC for caspase-3, Ac-IETD-AFC for caspase-8, and Ac-LEHD-AFC for caspase-9) were added. Finally, the plates were incubated at 37°C for 1 h and the caspase activity was measured using a microplate reader with the excitation and emission wavelengths at 380 and 440 nm, respectively.

### 2.15 Statistical analysis

All the data were expressed as mean ± SD, and the significant difference analysis was performed by *Tukey* multiple comparisons using SPSS Statistics 26.0 (SPSS Inc., Chicago, USA) with a significant level of *p* < 0.05.

## 3 Results and discussion

### 3.1 Formation of Cap@SeNPs

**Figure 1:**
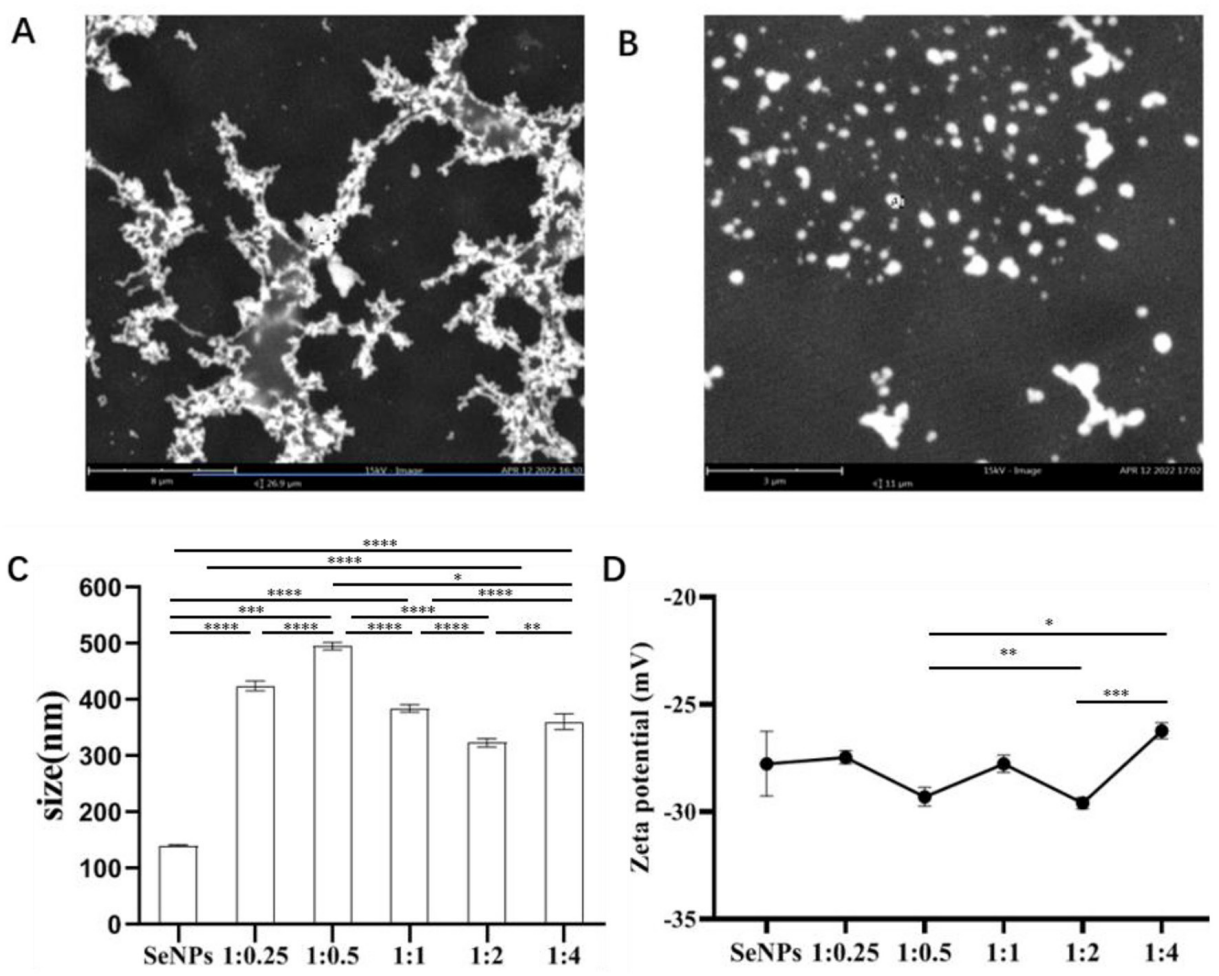
SEM images of SeNPs **(A)** and Cap@SeNPs **(B)**. Particles average size **(C)** and zeta potential **(D)** of Bc@SeNPs under different mass ratio of selenite and Cap. The elements composition of Cap@SeNPs. All data were expressed as mean ± SD, and the number of biological replicates was three (*n* = 3). *Indicated a statistical difference between the two groups (*P < 0.05, **P < 0.01, ***P < 0.001, ****P < 0.0001).

SeNPs coated with functional molecules can alter their zeta potential, enhance particle stability, and potentially modify the particle size. In this study, we found that cap is a functional component suitable for use as a surface modifier for SeNPs. Firstly, SeNPs were synthesized using the oxidation-reduction method according to the method reported by Tang et al. (29), and Cap@SeNPs were prepared by modifying SeNPs with Cap. The SEM image of SeNPs (Figure 1A) revealed the presence of inhomogeneous aggregates. However, after surface modification with Cap, the resulting products (i.e., Cap@SeNPs) were mostly spherical and more uniformly dispersed (Figure 1B), which suggested that Cap effectively prevented the aggregation of SeNPs, thus resulting in a homogeneous dispersion of the selenium nanoparticle system. The stability of nanoparticles is crucial for their potential use in future medical applications (6). Since the colloidal stability may be affected by the amount of Cap adsorbed on the SeNPs, we further examined the effect of the Cap concentration on the particle size and zeta potential of Cap@SeNPs. Figures 1C, D showed that the average particle size and zeta potential of the SeNPs were measured to be 141 nm and −27.8 mV, respectively. The optimum mass ratio of Na_2_SeO_3_ to Cap was found to be 1:2. At this ratio, the particle size and zeta potential of Cap@SeNPs were the smallest, measuring 324 nm and −29.6 mV, respectively. However, the particle size was still higher than that of SeNPs. This is inconsistent with the results of SEM. The possible reason is that the particle size of most SeNPs is too large, which exceeds the range of the instrument and cannot be identified, and finally only the data of the smaller particle size is detected. Zeta potential is correlated with the stability of colloidal dispersion, and the smaller zeta potential indicated the stronger electrostatic repulsion and ability to retain the monodispersity of nanoparticles (31). This further indicates that the particle size of Cap@SeNPs is smaller than SeNPs. From the above results, it was clear that Cap modification could improve the stability of SeNPs to a certain extent.

### 3.2 Characterization of Cap@SeNPs

**Figure 2:**
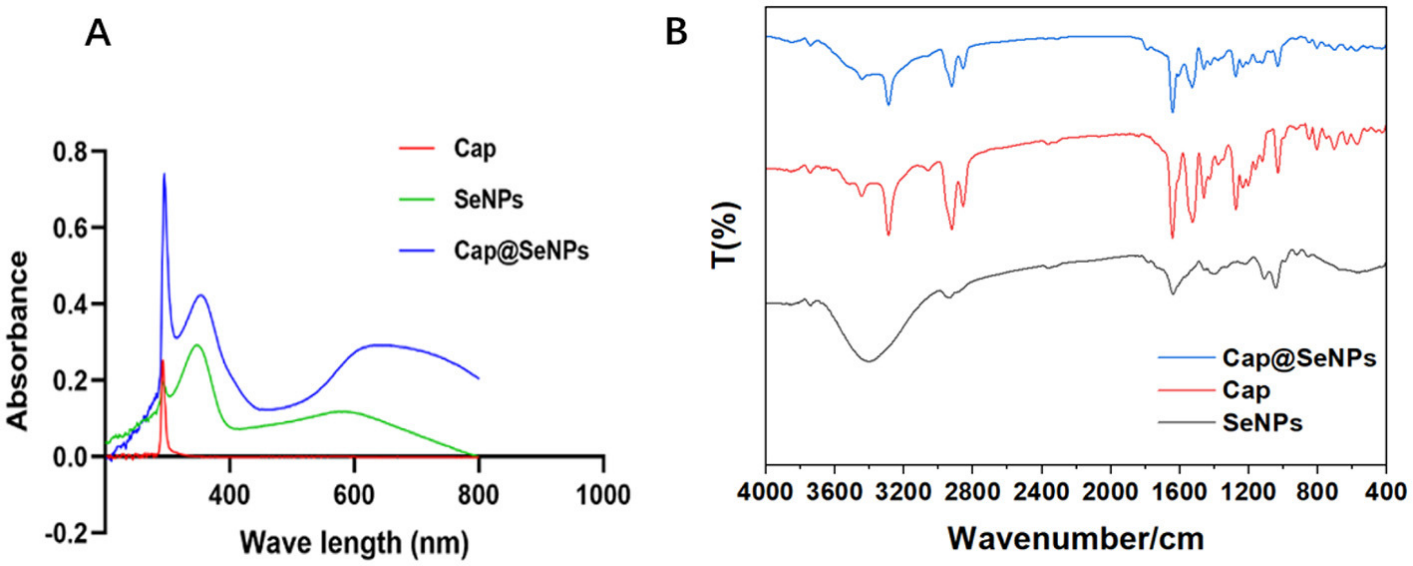
The UV-vis absorption spectrums of SeNPs, Cap, and Cap@SeNPs **(A)**. The FT-IR spectra of SeNPs, Cap, and Cap@SeNPs **(B)**.

UV-vis absorption spectra and FT-IR spectrum were used to identify the structure of the Cap@SeNPs. In the UV-vis absorption spectra (Figure 2A), Cap, SeNPs, and Cap@SeNPs showed common absorption peaks near 290 nm. Additionally, new absorption peaks appeared around 360 and 600 nm for SeNPs and Cap@SeNPs compared to Cap. The maximum absorption peak of Cap and Cap@SeNPs was around 290 nm, while that of SeNPs was at 360 nm. The strong absorption peak at 290 nm in the Cap@SeNPs spectrum is likely due to the existence of a benzene ring in Cap. In addition, all the characteristic absorption peaks of Cap@SeNPs were red-shifted compared to the spectrum of SeNPs, which could be due to the interaction of Cap with SeNPs. In the FT-IR spectrum shown in Figure 2B, the characteristic peaks of Cap@SeNPs were similar to those of Cap. Additionally, a new peak of 1,797 cm^−1^ was found in the FT-IR spectrum of Cap@SeNPs when compared with Cap. These results further demonstrated the successful combination of Cap with SeNPs. In the FT-IR spectra of Cap, the peaks at 3,500 to 3,200 cm^−1^ are attributed to stretching vibration of N-H and O-H, while the peaks at 1,609, 1,519, and 1,463 cm^−1^ correspond to the vibrational absorption of the benzene ring skeleton, moreover, the peaks at 1,643 and 1,033.91 cm^−1^ belong to stretching vibration of C=O and C-O-C, respectively (32–34). The appearance of the above characteristic peaks in the spectrum of Cap@SeNPs confirmed the modification of Cap on the surface of SeNPs. Therefore, the phenomena on UV-vis absorption spectra and FT-IR spectra indicated that Cap successfully bonded with SeNPs.

### 3.3 Anti-proliferation effect of nanoparticles on HepG2 cells

**Figure 3:**
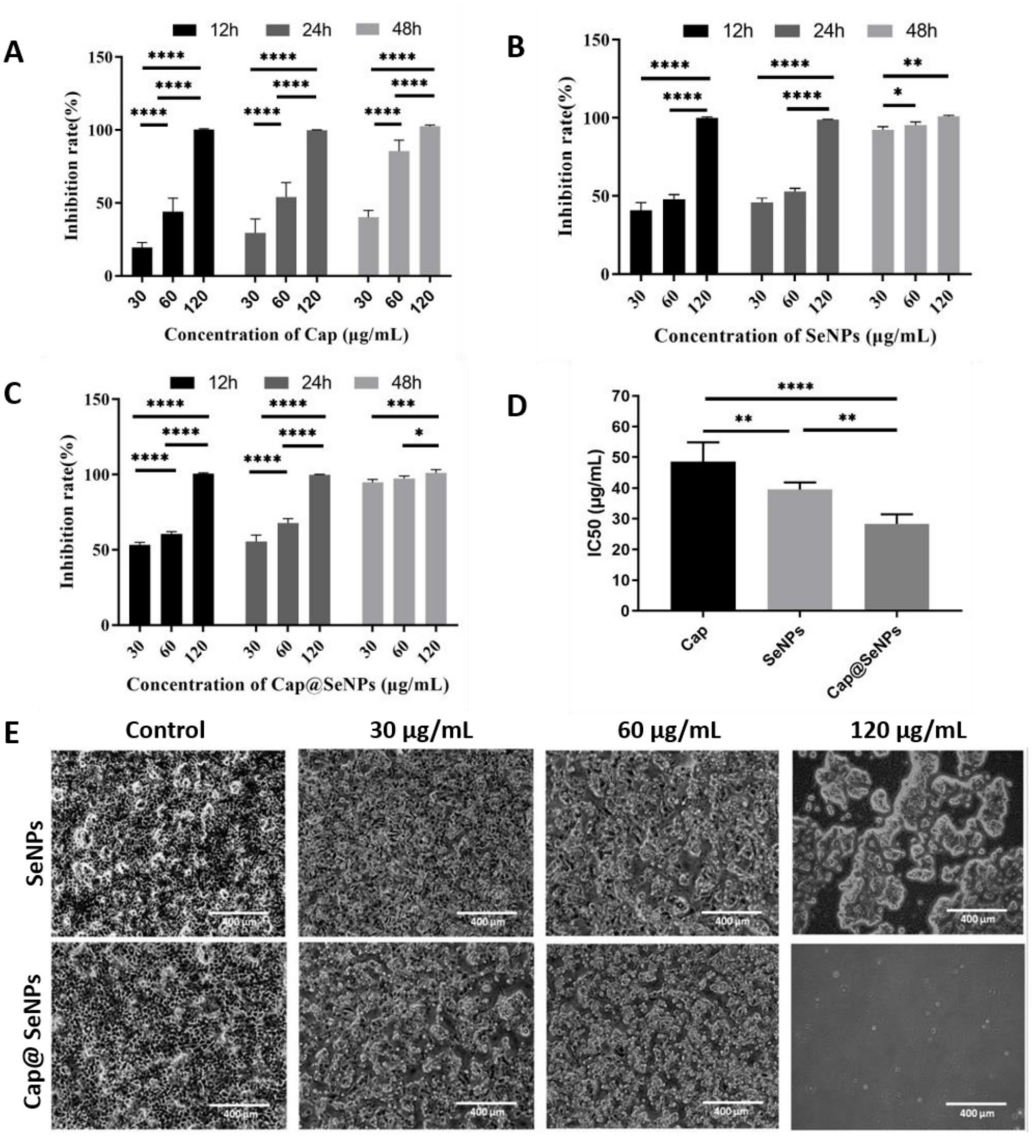
Anti-proliferation properties of Cap **(A)** SeNPs **(B)** and Cap@SeNPs **(C)** to HepG2 cells at 12, 24, and 48 h, respectively. **(D)** The IC50 of Cap, SeNPs, and Cap@SeNPs against HepG2 cells. **(E)** The morphologic observation of HepG2 after treatment of SeNPs and Cap@SeNPs for 24 h. All data were expressed as mean ± SD, and the number of biological replicates was three (*n* = 3). *Indicated a statistical difference between the two groups (*P < 0.05, **P < 0.01, ***P < 0.001, ****P < 0.0001).

HepG2 cells, originating from the liver tissue of individuals with hepatocellular carcinoma, are commonly used to evaluate the effectiveness of drugs against liver cancer *in vitro*. In the present study, HepG2 cells were exposed to three varying concentrations of SeNPs and Cap@SeNPs, and their anticancer effects were assessed through an MTT assay. As shown in Figures 3A–C, after treating HepG2 cells with different concentrations of Cap, SeNPs, and Cap@SeNPs, the inhibition rate of HepG2 cells gradually increased with the increase in both the duration of drug intervention and concentration. Compared to the blank control group, all concentrations of SeNPs and Cap@SeNPs exhibited significant inhibitory effects on the growth of HepG2 cells, with the inhibition rates showing a dose-dependent relationship over different time periods (*P* < 0.05). At the same drug concentration Cap@SeNPs demonstrated a higher inhibition rate against HepG2 cells than SeNPs and Cap in the intervention of 12 and 24 h. Furthermore, after 24 h of treatment with Cap, SeNPs, and Cap@SeNPs, the IC50 values were 48.6, 39.5, and 28.3 μg/mL, respectively (Figure 3D). Furthermore, the morphological observation of HepG2 cells treated with SeNPs and Cap@SeNPs for 24 h was conducted. As shown in Figure 3E, the untreated HepG2 cells appeared full and pike-shaped, while the cells treated with SeNPs or Cap@SeNPs became rounded and translucent with a reduced density of adherent cells. Taken together, Cap@SeNPs exhibited higher efficacy in antiproliferation, confirming that the surface modification with Cap not only significantly enhances their stability but also synergistically improves their bioavailability *in vivo*, thereby eliciting a stronger antiproliferative activity.

### 3.4 *In vitro* cytotoxicity of Cap@SeNPs

**Figure 4:**
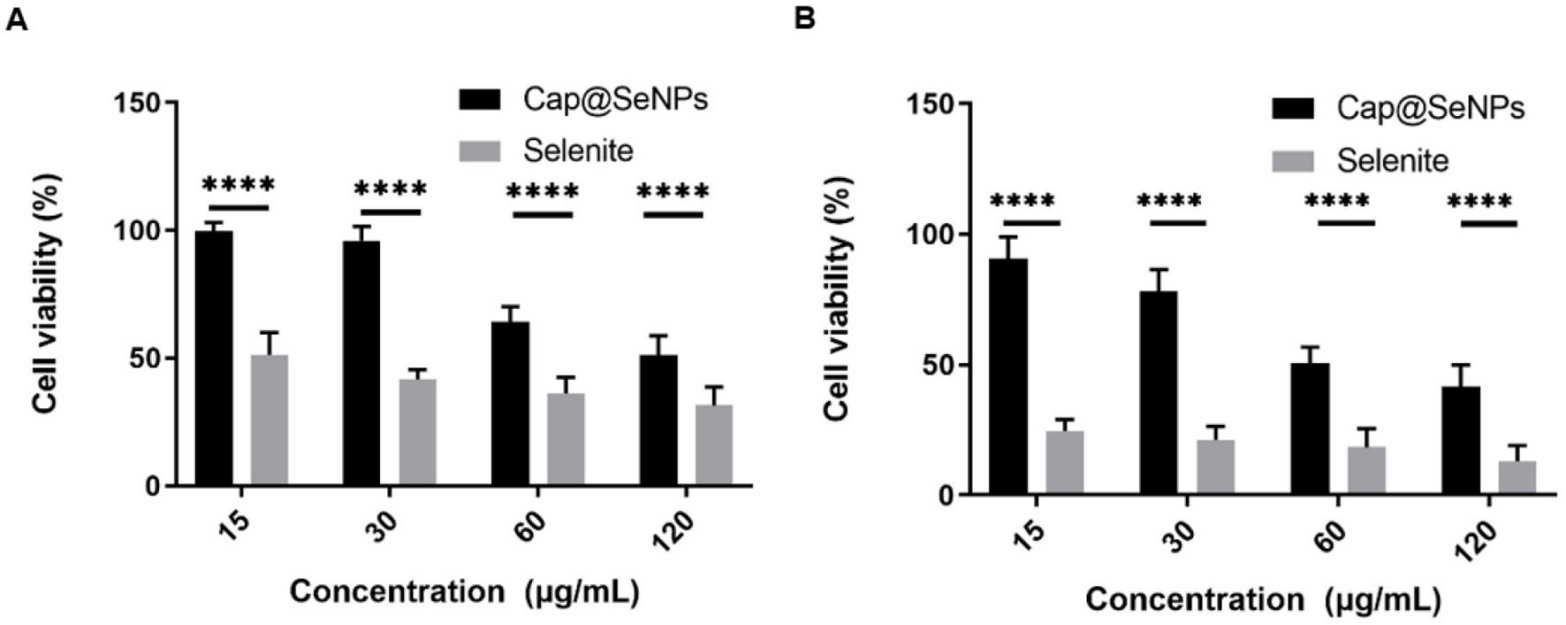
*In vitro* cytotoxicity of Cap@SeNPs in LO2 cells for 24 h **(A)** and 48 h **(B)**. All data were expressed as mean ± SD, and the number of biological replicates was five (*n* = 5). *Indicated a statistical difference between the two groups (****P < 0.0001).

The *in vitro* cytotoxicity of Cap@SeNPs and sodium selenite in the LO2 cell line was assessed and compared using the MTT method, with the results displayed in Figure 4. The findings revealed that low concentrations of Cap@SeNPs (15 and 30 μg/mL) exhibited cell survival rates exceeding 90% after a treatment of 24 h. Conversely, the cell survival rates for sodium selenite treatment were below 60% under identical doses and durations. Generally, regardless of the treatment duration being 24 or 48 h, both sodium selenite and Cap@SeNPs exhibited a dose-dependent inhibitory effect on cell growth. However, sodium selenite demonstrated a significantly stronger inhibitory effect compared to Cap@SeNPs. This discovery underscores that the cytotoxicity of Cap@SeNPs is markedly reduced in comparison to sodium selenite. In line with the outcomes of this study, previous research has reported analogous findings. For instance, Zhang et al. demonstrated that β-lactoglobulin-selenium nanoparticles can effectively mitigate the colon cytotoxicity of selenium (35). Moreover, Li et al. indicated that α-D-glucan-selenium nanoparticles can alleviate toxicity to liver cells (36). Additionally, Zhang et al. found in a mouse model that the acute toxicity of nano-selenium is seven times lower than that of sodium selenite (37), which is in accordance with the results of this study.

### 3.5 Cell cycle and apoptosis analysis

**Figure 5:**
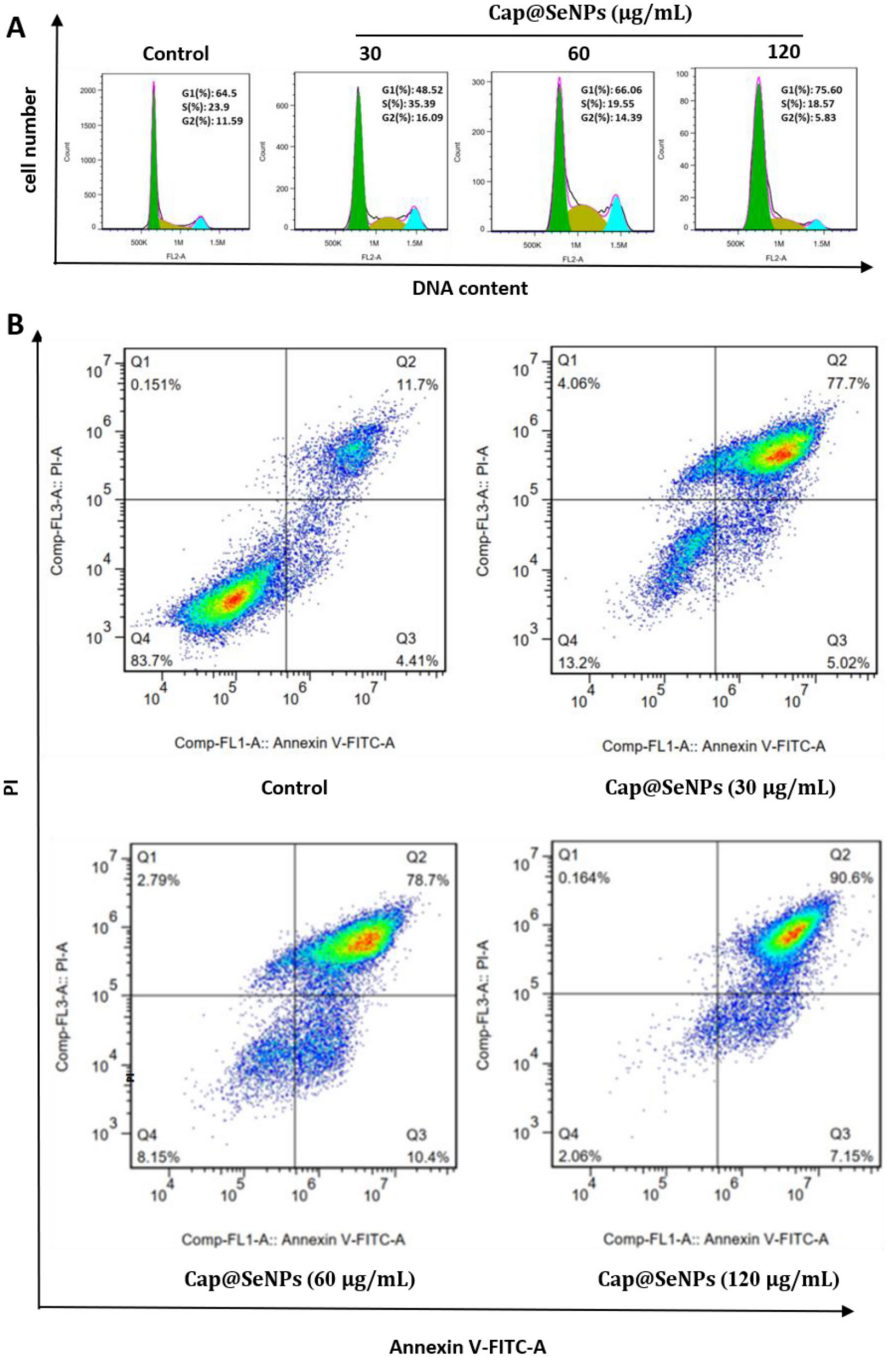
Effects of Cap@SeNPs on cell cycle distribution **(A)** and apoptosis **(B)** in HepG2 cells analyzed by flow cytometry.

Studies have indicated that the anti-proliferative effects of many chemicals on cancer cells are mediated by arresting the cell cycle and inducing apoptosis (38, 39). In this case, cell cycle and apoptosis analysis were performed to investigate the mechanism of the antiproliferative effect of Cap@SeNPs against cancer cells. HepG2 cells were treated with Cap@SeNPs for 24 h, and the cell cycle and apoptotic analysis were conducted using a flow cytometer. Figure 5A showed that 35.39% of HepG2 cells treated with 30 μg/ml of Cap@SeNPs were in the S phase, as compared with that of 23.90% in untreated cells. In addition, treatment of HepG2 cells with 120 μg/ml of Cap@SeNPs caused cell cycle arrest in the G1 phase with a decrease in S and G2 populations. For instance, the G1 population increased by 11.10%, while the S population and G2 population decreased by 5.33 and 5.79%, respectively in HepG2 cells treated with 120 μg/ml of Cap@SeNPs, as compared with the untreated cells. However, the Cap@SeNPs treatment of 60 μg/ml had no effect on the cell cycle. The above results suggested that Cap@SeNPs have no relevance to the cell cycle, and the inhibition of cell proliferation by Bc@SeNPs may be achieved by the inducing apoptosis. Therefore, the effect of Cap@SeNPs treatment on apoptosis in HepG2 cells was also detected by Annexin-V/PI double staining in this work. The apoptosis rate of HepG2 cells gradually increased with the growing concentration of Cap@SeNPs (Figure 5B). The percentage of apoptotic cells increased to 82.72, 89.10, and 97.75% after the treatment of HepG2 cells with 30, 60, and 120 μg/mL of CAP@SeNPs, respectively, compared to 16.11% in untreated cells. This demonstrated that Cap@SeNPs inhibited the proliferation of HepG2 cells by promoting apoptosis.

### 3.6 Elevation of intracellular reactive oxygen species levels induced by Cap@SeNPs

**Figure 6:**
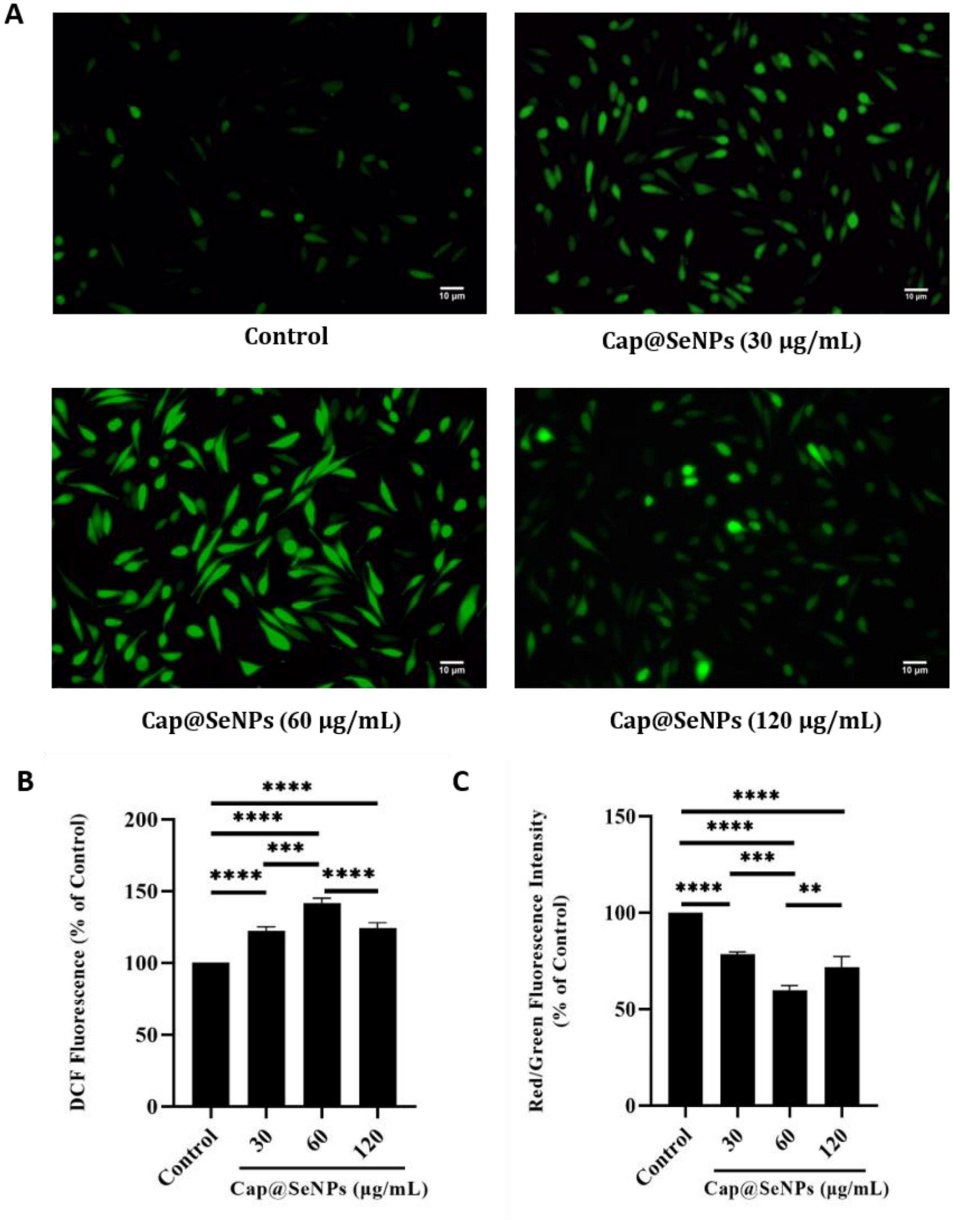
Effects of Cap@SeNPs on ROS levels **(A)** in HepG2 cells recorded by fluorescence microscopy. Effects of Bc@SeNPs on ROS levels **(B)** and Δψm **(C)** in HepG2 cells examined by a microplate reader. All data were expressed as mean ± SD, and the number of biological replicates was three (*n* = 3). *Indicated a statistical difference between the two groups (**P < 0.01, ***P < 0.001, ****P < 0.0001).

Intracellular ROS levels are closely related to apoptosis (40). The oxidative-antioxidant system in organisms can maintain cellular ROS levels within a stabilized range. However, overproduction of ROS can prompt a series of programmed cellular responses such as apoptosis and autophagy (41). In this study, HepG2 cells were stained with DCF-DA dye, and the intracellular ROS levels were subsequently detected using a microplate reader and a fluorescence microscope. After treatment with Cap@SeNPs of 30, 60, and 120 μg/mL for some time, the relative intracellular ROS levels of HepG2 cells were significantly increased by 21, 30, and 4%, respectively, compared to the untreated cells (Figure 6B). Furthermore, the green fluorescence intensity was also enhanced following the treatment of Cap@SeNPs (Figure 6A). The treatment of HepG2 cells with 60 μg/mL of Cap@SeNPs resulted in the highest intracellular ROS level and green fluorescence intensity. The above results indicate that Cap@SeNPs elevate the ROS levels in HepG2 cells, and the highest was observed at the concentration of 60 μg/mL.

### 3.7 Δψm loss induced by Cap@SeNPs

**Figure 7:**
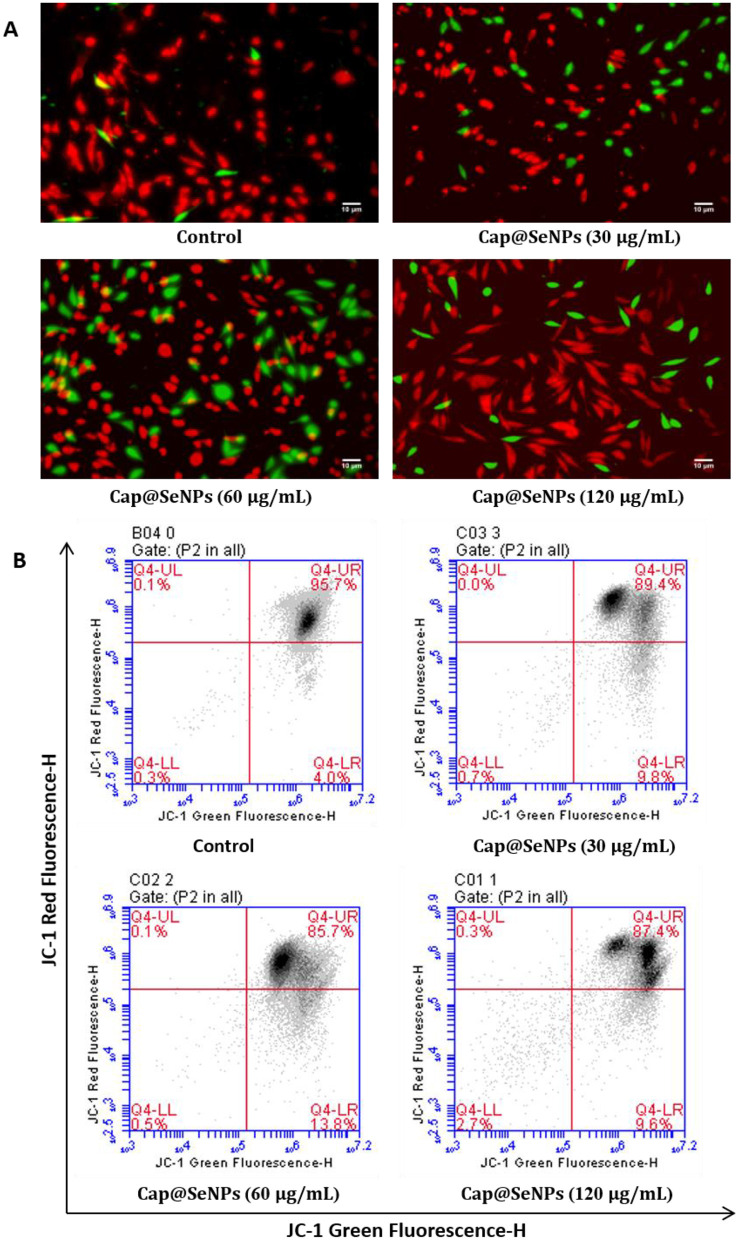
Effects of Cap@SeNPs on Δψm **(A)** in HepG2 cells recorded by fluorescence microscopy. The Δψm in HepG2 cells induced by Bc@SeNPs recorded by flow cytometer **(B)**.

Mitochondria are important organelles in the apoptosis pathway (42). Mitochondrial dysfunction and a decrease in Δψm are critically associated with apoptosis (43). JC-1 is a fluorescent probe widely used to detect Δψm, which is potential-dependent and usually accumulates in mitochondria (44). When the Δψm is high, JC-1 polymerizes under the influence of potential energy and emits red fluorescence. In apoptotic cells, however, JC-1 exists as a monomer due to the decreased Δψm and emits green fluorescence. In this study, HepG2 cells were stained with JC-1 dye, and the intensity of red and green fluorescence was subsequently detected using a microplate reader, afluorescence microscope, and a flow cytometer to analyze the changes of Δψm in HepG2 cells. As shown in Figure 7A, CAP@SeNPs increased the intensity of green fluorescence and decreased the red/green (R/G) fluorescence intensity ratio (Figure 6C), indicating mitochondrial depolarization. Additionally, treatment of HepG2 cells with 60 μg/mL of Cap@SeNPs resulted in the highest intensity of green fluorescence and the lowest R/G value. Compared with untreated cells (4.0%), the intensity of green fluorescence increased to 9.8, 13.8, and 9.6% after treating HepG2 cells with 30, 60, and 120 μg/mL of CAP@SeNPs, respectively, suggesting a decrease in Δψm (Figure 7B). The above results demonstrate that CAP@SeNPs induce mitochondrial dysfunction in HepG2 cells, which is associated with the apoptosis of these cells.

### 3.8 Condensation of chromatin in HepG2 cells induced by Cap@SeNPs

**Figure 8:**
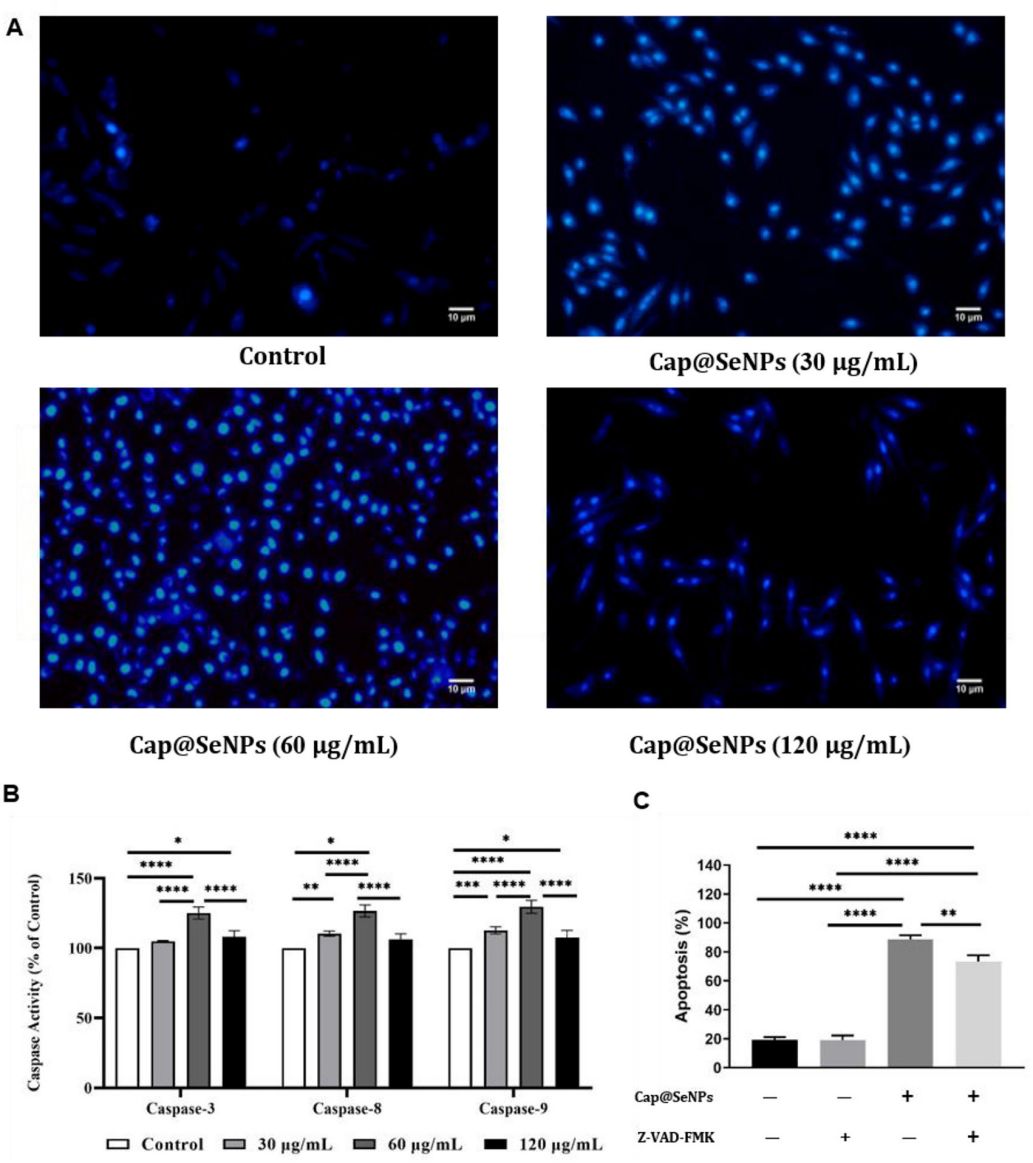
Effects of Cap@SeNPs on the degree of chromatin condensation recorded by fluorescence microscopy **(A)** and the activities of caspase proteases (including caspase-3, caspase-8, and caspase-9) examined by a microplate reader **(B)** in HepG2 cells. The effect of caspase inhibitors on cell apoptosis induced by Cap@SeNPs **(C)**. All data were expressed as mean ± SD, and the number of biological replicates was three (*n* = 3). *Indicated a statistical difference between the two groups (*P < 0.05, **P < 0.01, ***P < 0.001, ****P < 0.0001).

Chromatin pyknosis is a characteristic feature of cell apoptosis (45). To further evaluate Cap@SeNPs-induced apoptosis, HepG2 cells were stained with Hoechst 33258 dye and observed using a fluorescence microscope. Hoechst 33258, a non-embedded blue fluorescent dye, is biofilm-permeable and selectively binds to nuclear DNA regions enriched in A-T base sequences to color the nucleus. Apoptosis is accompanied by increased membrane permeability, which allows more Hoechst to enter the cells (46). Furthermore, due to chromatin condensation, the pyknotic nucleus can exhibit a high degree of blue fluorescence after staining with Hoechst. As shown in Figure 8A, untreated cells emitted low blue fluorescence, whereas the nuclei of HepG2 cells treated with different concentrations of Cap@SeNPs emitted bright blue fluorescence. Moreover, HepG2 cells treated with 60 μg/mL of Cap@SeNPs showed the highest blue fluorescence intensity. The above results demonstrate that CAP@SeNPs can suppress cell proliferation by inducing chromatin condensation, and the greatest nuclear chromatin condensation was observed in HepG2 cells treated with 60 μg/mL of CAP@SeNPs.

Therefore, CAP@SeNPs-induced apoptosis in HepG2 cells may be associated with ROS-mediated oxidative stress, mitochondrial dysfunction, and nuclear chromatin sequestration.

### 3.9 Apoptotic signaling pathway activated by Cap@SeNPs

Caspase proteases play a critical role in the mediation of cell apoptosis (47). The caspase proteases consist of caspase-3, caspase-8, and caspase-9, of which caspase-3 is the most essential effector element in the apoptotic pathway (48). Figure 8B revealed that the activities of caspase-3, caspase-8, and caspase-9 were increased after treatment with different concentrations of CAP@SeNPs. Among them, 60 μg/mL of CAP@SeNPs resulted in the maximum enzyme activities of caspase-3, caspase-8, and caspase-9. To identify the role of caspases in CAP@SeNPs-induced apoptosis, the caspase inhibitor Z-VAD-FMK (a general caspase inhibitor) was utilized to examine its effects on apoptotic cells. As shown in Figure 8C, CAP@SeNPs at a concentration of 60 μg/mL significantly induced apoptosis, which was inhibited upon the addition of Z-VAD-FMK. These results demonstrated that CAP@SeNPs can induce apoptosis in HepG2 cells by activation of the caspase protease family.

Apoptosis is a pivotal pathway in cancer cell death, with the mechanism involving reactive oxygen species (ROS) and membrane potential alterations being particularly notable (49). Studies have shown that ROS accumulation within cells disrupts mitochondrial function, leading to a decrease in membrane potential. This sequentially triggers apoptotic signaling pathways, including the release of cytochrome C and the activation of caspase enzymes, which ultimately leads to cell apoptosis (50, 51). Our research has revealed that Cap@SeNPs treatment induces chromatin condensation and apoptosis in HepG2 cells, hinting that the anti-proliferative effect of Cap@SeNPs is predominantly mediated through the induction of apoptosis.

Delving deeper into the apoptotic mechanism of Cap@SeNPs, it is found that Cap@SeNPs cause a reduction in mitochondrial membrane potential in HepG2 cells, resulting in mitochondrial dysfunction. Additionally, Cap@SeNPs was found to activate caspases-3,−8, and−9. Based on these findings, we deduce that Cap@SeNPs elevate ROS levels in HepG2 cells, which in turn leads to a decrease in ΔΨm, thereby triggering mitochondrial dysfunction. Consequently, this chain of events initiates the cleavage and activation of Caspases-3,−8, and−9, ultimately promoting the apoptotic process in HepG2 cells. Our findings resonate with those of Tang et al., who reported that Betacyanins-SeNPs exhibit potent antitumor effects *in vitro* by modulating ROS and mitochondrial membrane potential, further activating the caspase cascade and inducing mitochondria-mediated cell apoptosis (29). Notably, it is observed that cellular ROS levels, mitochondrial membrane potential, and caspase protease activity peaked after treatment with 60 μg/mL Cap@SeNPs in this research. However, the highest cell death rate was achieved with 120 μg/mL Cap@SeNPs. A conceivable reason for this discrepancy is that 120 μg/mL of Cap@SeNPs may induce cell death not only through caspase-dependent apoptosis but also via other pathways, such as necroptosis and autophagy (52–54). Nevertheless, this hypothesis necessitates further investigation in future studies. Additionally, the *in vivo* toxicity, anti-proliferative activity, and underlying mechanisms of Cap@SeNPs require further extensive exploration. Furthermore, it is advisable for future research initiatives to employ targeted strategies that accurately guide Cap@SeNPs to specific cell types or tissues, ultimately leading to an increase in both the bioavailability and therapeutic effectiveness of Cap@SeNPs.

## 4 Conclusion

We have prepared nanoparticles with good stability and dispersion by surface modification of SeNPs using Cap. Cap@SeNPs can exert anti-proliferative effects on HepG2 cells by arresting the cell cycle and inducing apoptosis. Treatment with Cap@SeNPs increases intracellular ROS levels in HepG2 cells and subsequently induces mitochondrial damage, which activates caspase proteases, leading to chromatin condensation and ultimately apoptosis of HepG2 cells. Thus, our study suggests that Cap@SeNPs can be used as a potential chemotherapeutic agent for cancer therapy.

## Data Availability

The raw data supporting the conclusions of this article will be made available by the authors, without undue reservation.
